# A standardised low-cost membrane blood-feeder for *Aedes aegypti* made using common laboratory materials

**DOI:** 10.7717/peerj.14247

**Published:** 2022-10-28

**Authors:** Peter A. Faber, Ashritha J.A.P.S. Dorai, Steven L. Chown

**Affiliations:** School of Biological Sciences, Monash University, Melbourne, Victoria, Australia

**Keywords:** *Aedes aegypti*, Membrane feeding, Bloodfeeding, Artificial blood feeding

## Abstract

Blood feeding is a necessary part of laboratory studies involving mosquitoes and other hematophagous arthropods of interest in medical and ecological research. However, methods involving hosts may present serious risks, require ethics approvals and can be expensive. Here we describe an insect blood feeder made using common laboratory materials, which is low cost (<US$100) and can be constructed and operated with little technical expertise. We compared the blood feeder containing an artificial blood diet, Skitosnack, to direct human arm feeding for *Aedes aegypti* (Diptera: Culicidae), in terms of engorgement rate, fecundity and hatch rate. No significant difference in fecundity between the two approaches was found, (mean ± SD); direct human arm: 56 ± 26 eggs/female, artificial method: 47 ± 25 eggs/female, *P* = 0.569. Engorgement rates (direct human arm: 97.8 ± 4%, artificial: 64.1 ± 23%, *P* < 0.05) and hatch rates (direct human arm: 75 ± 12%, artificial: 59 ± 14%, *P* < 0.05) were lower in the artificially fed mosquitoes. Despite these differences, we maintained a healthy mosquito colony for 10 generations using the artificial feeding approach. Results from this comparison are within the range of other studies which compared direct host feeding with an artificial feeding method. We anticipate that the blood feeder presented here could substantially reduce costs usually required to establish a standardised and effective blood feeding method for maintaining mosquito colonies or conducting experiments, extending the capability of laboratories especially where research resources are limited, but vector-borne diseases common.

## Introduction

Studies on mosquitoes have become increasingly important due to the persistent, high burden of vector-borne diseases, such as dengue and malaria (Gubler, 1998; WHO, 2018) and the unpredictable emergence of other arboviruses in recent decades, creating global health concerns (Gubler, 2002; Musso et al., 2018). Many medically important species of the mosquito genera *Aedes*, *Culex* and *Anopheles* are routinely used in laboratory-based trials to understand life history components, disease dynamics, the efficacy of vector control treatments, and as model organisms to investigate functional genetics (Haugen et al., 2011), learning behaviour (Baglan, Lazzari & Guerrieri, 2017; Vinauger et al., 2018), and sperm biology (Degner & Harrington, 2016) among others (Clemons et al., 2010; Schmidt-Ott & Lynch, 2016). The specialisations of mosquitoes, such as their highly sensitive sensory systems, host seeking and blood feeding behaviours, may help to answer research questions which are unable to be asked of other common model organisms such as *Drosophila melanogaster* (Matthews & Vosshall, 2020).

The females of all medically important species of mosquito require a blood meal to develop and lay eggs. Perhaps the most complicated and difficult to simulate step of rearing mosquitoes in the laboratory is this process of blood feeding. Traditional methods, such as using the forearm of an entomologist or willing volunteer, may present risks, for example, if the mosquito colony or person feeding them is unknowingly infected with a pathogen (Alves et al., 2005; Duong et al., 2015). For instance, if colonies are created with first generation mosquitoes from field collections, they may have inherited arboviruses via vertical transmission (Lequime, Paul & Lambrechts, 2016) or as larvae developing in water contaminated with the waste of infected persons (Du et al., 2019).

The ethical considerations of blood feeding laboratory or semi-field mosquito colonies on human volunteers are not always clear and the activity may be ethically questionable, for instance, when research institutions use junior staff for the activity (Ndebele & Musesengwa, 2012; Harrington, Foy & Bangs, 2020). Currently, institutions may require ethics approval for the use of researcher or volunteer blood feeding or it may be considered an occupational risk (Achee et al., 2015; Harrington, Foy & Bangs, 2020). The risks are increased if the laboratory concurrently conducts work with live pathogens or is located in a region with endemic pathogen transmission which could be unwittingly introduced into colonies. As mosquito borne diseases such as dengue and malaria are widespread throughout the tropics (Feachem et al., 2010; Brady et al., 2012), the many research institutions located in these regions require ongoing vigilance if they conduct blood feeding, such as regular pathogen screening of volunteers and vector colonies, stringent laboratory rules and containment practises to minimise the risk of accidental pathogen exposure (Knols et al., 2002; Harrington, Foy & Bangs, 2020).

To obviate the need for feeding on humans, alternatives such as direct feeding on animals are often used. Using animals such as mice, rats, chickens, rabbits, guinea pigs, cattle, hamsters or pigeons for feeding mosquitoes may cause them pain, distress and/or discomfort (Edman & Scott, 1987) and usually requires animal ethics approval from research institutions (Benedict & Dotson, 2007). In practise, these methods often require animal preparation such as hair or feather removal, restraint and anaesthetics, complicating the process (Bailey et al., 1978; Foster, 1980). Rearing and maintaining animals in adherence with local regulations for the purposes of blood feeding may be expensive and require specialised training and qualifications (Services, 2015; Baughman et al., 2017). Modern research involving animals necessitates the consideration of the three R’s principle—replacement, reduction and refinement; therefore, alternatives for blood feeding hematophagous arthropods are desirable where live animals can viably be substituted (Costa-da Silva et al., 2014). In certain settings, replacement of animals with alternative methods may be difficult due to limited resources (Nyika, 2009), indicating a need for the development of methods and protocols which are more broadly accessible.

Many successful attempts have been made to replicate ectoparasite blood feeding in the laboratory (Romano et al., 2018), but many also suffer from a lack of standardisation, excessive cost, difficult manufacturing, or the requirement of ongoing maintenance during the blood feeding process to maintain a blood temperature warm enough to elicit strong feeding responses. Commercial options such as electronically heated Hemotek (UK) (Hemotek, 2020) based on (Cosgrove et al., 1994), and water heated glass membrane feeders (Chemglass; (Sciences, 0000)) based on (Rutledge, Ward & Gould, 1964) are available and commonly used for infection assays and colony maintenance, but are expensive and only available from a limited number of suppliers which may make them inaccessible for some laboratories. Recently, 3D printed membrane feeders for mosquitoes have been made (Witmer et al., 2018; Graumans et al., 2020), but the 3D printing needs to be of sufficient quality to be impermeable to water and blood and needs to be easily cleaned and in some situations, sterilised. Unless specific materials are used, a 3D printed blood feeder would be unable to be sterilised at high heat. Currently, the technology is unavailable in some places, where low-tech options would be favoured.

To avoid these disadvantages, we have developed a standardised and adaptable blood feeding method for *Aedes aegypti* which can be constructed from common laboratory materials at relatively low cost (<$100 USD), and trialled it using an artificial diet “SkitoSnack” (Gonzales et al., 2018) which consists of bovine serum albumin as a protein source, bovine haemoglobin, egg yolk powder, glucose, adenosine triphosphate and a bicarbonate buffer containing chloride salts of sodium, potassium, calcium and magnesium.

*Aedes aegypti* has a strong preference for human hosts and therefore, artificial methods are expected to be less effective than directly feeding on a human arm (Baughman et al., 2017). The magnitude of this effect is likely influenced by many variables and between studies observing this comparison, methods vary widely. However, since replacing hosts with artificial methods can provide multiple advantages, and can provide a means for colony maintenance, experiments and assays, it remains a desirable option for many, and should be given consideration given the complexities of the alternatives.

## Materials and Methods

### Blood feeder description

**Figure 1:**
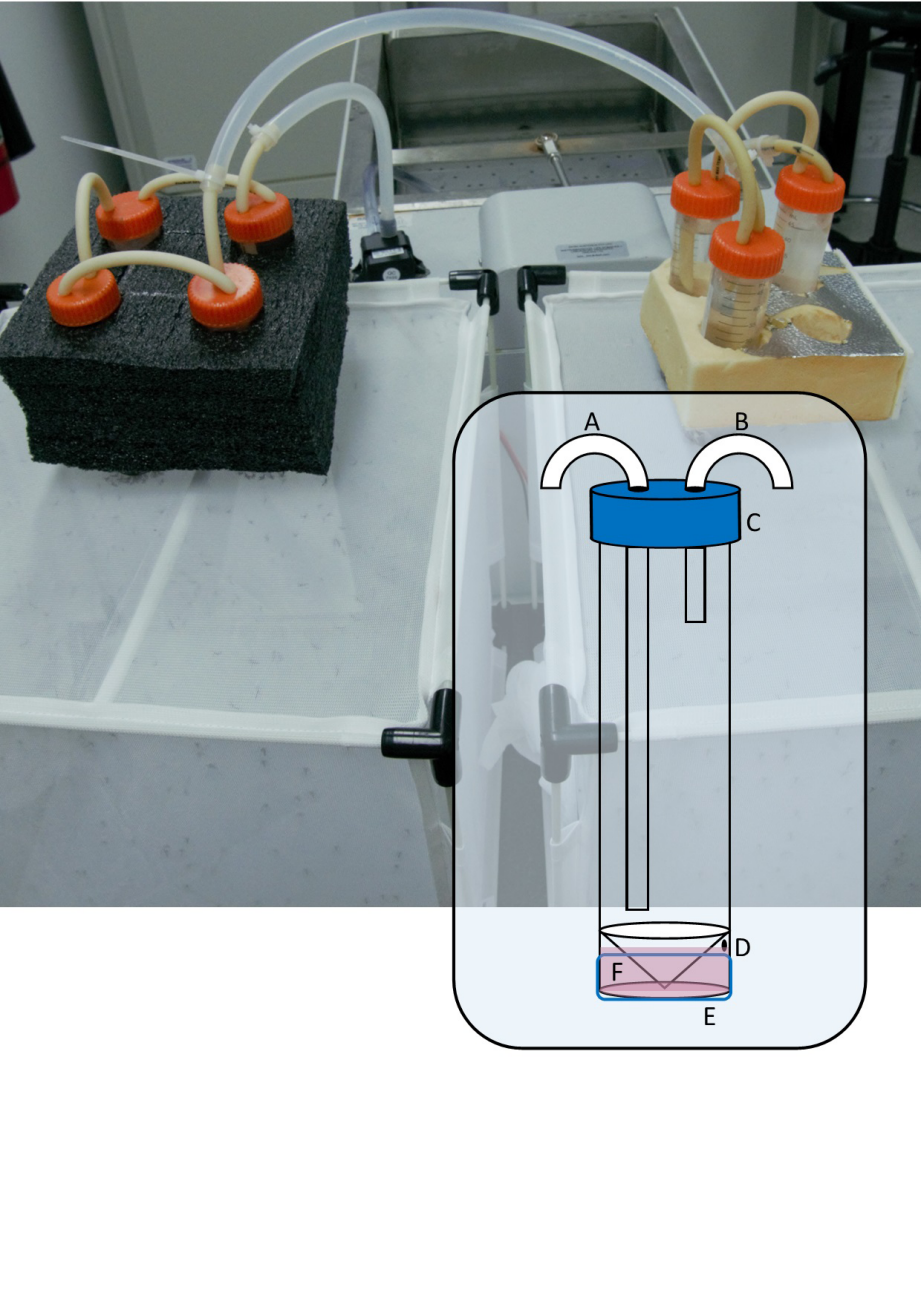
Photograph of seven blood feeder units in series, feeding two colonies. Inset: Diagram of modified 50 mL skirted centrifuge tube showing (A) warm water inlet tube, (B) outlet tube, (C) cap with 2 × 5 mm holes, (D) 1 mm filling port, (E) stretched Parafilm ‘M’ membrane, (F) filled reservoir.

The major components of the blood feeding system are (Fig. 1): (1) A 50 mL skirted/self-standing centrifuge tube (Capp, Nordhausen, Germany). The plastic skirt at its base becomes the reservoir for blood or blood substitute, holding approximately 3 mL. Near the top of the skirt, a 1 mm hole is drilled through to the reservoir which serves as the filling port. In the cap, two 5 mm holes are drilled to accommodate the water tubing. (2) Soft silicon tubing (Masterflex, PharMed BPT, Vernon Hills, USA), 3.1 mm ID, cut to size and forced through the 5 mm holes in the cap, creating a leak-proof seal. (3) A 30 mm length of 2 inch Parafilm ‘M’ stretched to double its dimensions. (4) A water bath and pump. We tested the system with a WS17-2 laboratory water bath (Sheldon Manufacturing Inc, Cornelius, USA) as well as a low-budget in-house designed water bath created using an ITC-308 temperature controller relay (Inkbird, Shenzhen, China) and a 2000 W immersion element (unbranded), immersed in a bucket of water. In both cases, water was circulated with a 12V DC brushless 4.8 W aquarium or fountain pump (see Video S1 for a video detailing construction) and could heat multiple feeding units at once.

Warm water is circulated through the centrifuge tube which transfers heat to the reservoir through its plastic base, maintaining the membrane temperature at 37 °C according to a non-contact infrared thermometer. Other ways to circulate warm water through the unit are likely possible, depending on the equipment available and the researcher’s ingenuity.

The system is modular and rests on top of the mesh of an enclosure containing female mosquitoes. We used packaging foam featuring ∼28 mm holes to maintain the feeder units upright and ensure good contact with the mesh of a 32.5 × 32.5 × 32.5 cm insect rearing cage (Bugdorm, MegaView Science Co. Ltd., Taichung, Taiwan) containing our main *Aedes aegypti* colony as well as our experimental replicates in 500 mL plastic cups lightly scratched on the inside with sandpaper and covered in bridal tulle secured with an elastic band. In series, the system can be used to blood feed numerous small colonies or experimental replicates simultaneously. For maintaining the colony used in the trials described below, we connected three or four modules together because the colonies typically contained 300–400 females and the surface area of the membrane is 5.3 cm^2^ which determines the number of individuals that can feed per unit at one time. For the validation experiment, we connected three units in series so that we could feed three replicate cups of female mosquitoes simultaneously.

### Validation

We used a wild type line of *Aedes aegypti* originally collected from Townsville, QLD, Australia. Artificial feeding began on the 9th generation and prior to this, the colony was maintained by direct human arm feeding. The experiment to quantify blood feeding success of the artificial method commenced with the 11th generation, after two generations of artificial feeding. Following (Ross et al., 2017) with modifications, larvae were reared by hatching eggs in N_2_ purged deionised water and fed on AquaOne Vege Wafers fish food (Aqua Pacific, Southampton, UK)) in covered plastic trays at 27 °C and 12:12 photoperiod until pupation. Pupae were pipetted into 500 mL cups with 250 ml of deinonised water and placed into a Bugdorm cage (32.5 × 32.5 × 32.5 cm). Adults were maintained on 10% w/w sucrose solution and kept at 27 °C, 80% RH and 12:12 photoperiod until required for experiments. They were then aspirated from the cage, anaesthetised under CO_2_, sorted and counted into 500 mL cups. Before blood feeding, the adult mosquitoes were starved of sucrose for at least 16 h. Fifteen six-day old adult female mosquitoes were provided with a blood meal from either the blood feeding unit containing SkitoSnack for 2 h, or the researcher’s (PAF) arm for 1 h, in a laboratory at constant temperature of 25 °C. One hour was deemed to be a sufficient period for mosquitoes to feed completely using the direct feeding method, however extra time was given for the artificial method which evidently lacked some cues for blood feeding and therefore elicited slower feeding responses in the mosquitoes. The forearm blood feeding was conducted by PAF under the advice of Monash University Human Ethics Committee. Each treatment was conducted in triplicate and the experiment was repeated on four generations resulting in a sample size of 12 groups of 15 female mosquitoes for all measures. For both blood feeding methods, we determined engorgement rate (%), fecundity (eggs/female) and egg hatch rate (%) as widely accepted measures of blood feeding performance (Ross, Lau & Hoffmann, 2019; Paris et al., 2018).

Engorgement rates were determined after blood feeding by anaesthetising the mosquitoes with carbon dioxide and counting the number of fully and partially engorged females. Unfed females were removed so that only engorged females were allowed to lay. Moist 90 mm No. 2 filter paper (Advantec Toyo Kaisha Ltd., Tokyo, Japan) partly submerged in a small cup of 30 mL of deionised water was provided as an oviposition substrate for five days after blood feeding, after which the egg papers were removed and dried slowly to near-dryness for four days between layers of paper towel, absorbent cloth and glass sheets and stored (following O’Neill et al., 2018). Fecundity was measured by photographing egg papers and counting the number of eggs using the Multi-point tool on ImageJ (Schneider, Rasband & Eliceiri, 2012). The average number of eggs laid per engorged female was then calculated for each replicate. Hatch rate was measured by counting and hatching approximately 100 eggs from each replicate in deionised water at 27 °C, with a small amount of larval diet, followed by counting hatched larvae and determination of the hatch rate as a percentage.

Statistical analysis was conducted in R version 3.5.0 (R Core Team, 2018). Generalised linear models were fitted on the engorgement rates, hatch rates and fecundity (Crawley, 2012) to test the hypotheses that these measures differed significantly between direct and artificial methods.

As the approaches used to investigate artificial feeding methods vary widely between studies, *e.g.*, (Deng et al., 2012; Costa-da Silva et al., 2013; Tan et al., 2016; Gunathilaka et al., 2017), we chose to investigate the general effect of substituting a direct host method with an artificial method, which is a frequently stated general goal. For comparisons with previous investigations using a variety of different feeding methods, Google Scholar and Scopus databases were searched using the terms: membrane+aedes+aegypti and artificial+blood+feeding+aedes+aegypti. Titles, abstracts and methods sections were screened to identify primary literature conducting a direct host feeding method alongside an artificial membrane feeding method for *Aedes aegypti*. The reference lists of these studies were also investigated, screened and any further studies identified. Records not comparing the direct host and artificial methods for any of three measures; engorgement rate, fecundity and hatch rate were excluded.

Where possible, means, standard deviations and samples sizes were extracted or calculated from the text or from figures using the R package *metaDigitise* (Pick, Nakagawa & Noble, 2019). Where there were multiple comparisons made in a study, the simplest comparison was chosen or subgroups were pooled. Where measures of variation around the mean other than standard deviation were given, such as standard error or confidence intervals, standard deviations were calculated according to the equations outlined in the Cochrane handbook for systematic reviews of interventions (Higgins et al., 2019). Several studies had means followed by a “±” value without specifying whether this was standard deviation or standard error, and this was assumed to be standard error based on similar works. Several papers had no indication of variability at all and these were excluded.

Using the extracted means, standard deviations and sample sizes, Cohen’s d effect sizes with Hedges’ correction were calculated using equations from (Hedges, 1981) and code from (Hamman et al., 2018). Confidence intervals (0.95) for effect sizes were calculated using the R package *MBESS* (Kelley, 2007; Kelley, 2015) and weights based on the precision of each estimate were calculated as the reciprocal of the variance in the calculated effect size.

## Results

**Figure 2:**
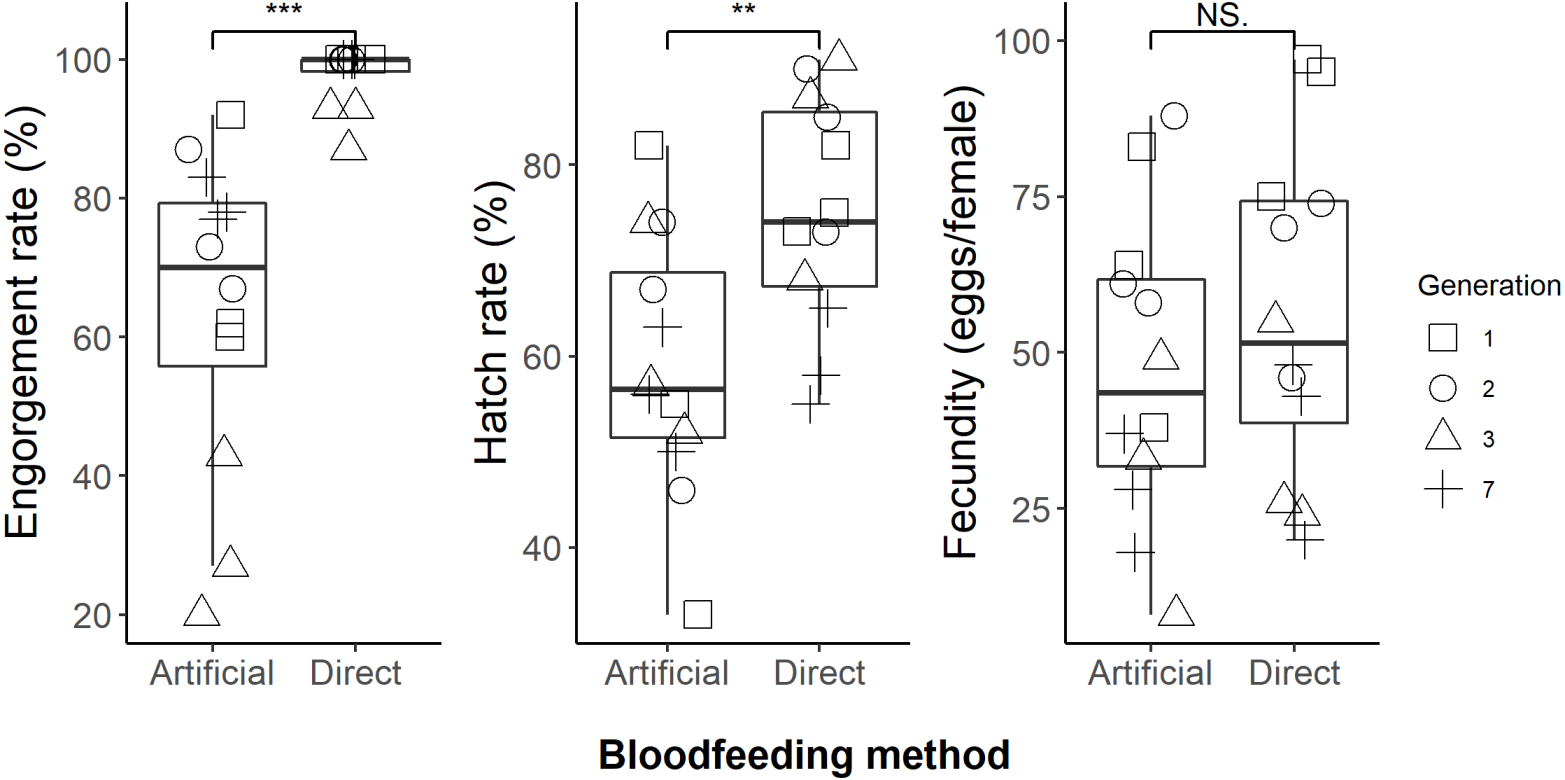
Comparison of direct human arm feeding and artificial feeding for engorgement rates, hatch rate and fecundity (eggs per engorged female). Boxplots show the median, interquartile range and the range of data. Significant differences between methods are represented on the plots and significance tests and *P* values are shown in Table 1.

We successfully maintained our *Aedes aegypti* colony using the artificial blood feeding method for ten generations, to provide mosquito larvae for unrelated experiments. In our blood feeding experiments, there was high variability in all measures, with the exception of engorgement rate for the human arm fed treatment. Engorgement rates (Fig. 2) were generally high for mosquitoes provided with the human forearm (mean ±standard deviation: 97.8 ±  4.3%); significantly higher (Table 1) than those fed using the artificial method (64.1 ±  23.2%). Fecundity (Fig. 2) was highly variable for both treatments, 56.1 ±  26.4 eggs per engorged female for human forearm and 47.1 ±  24.7 for the artificial method and this was not significantly different (Table 1). Engorgement rate and fecundity was lower in the third generation, but we think this was an experimental error relating to delayed and asynchronous larval development and therefore the age of the experimental adult mosquitoes. This effect was observed in both the direct host fed and the artificially fed treatments and contributed to the variability seen overall in these two measures. The hatch rates (Fig. 2) were 75.2 ±  12.1% for the human forearm which was significantly higher (Table 1) than the artificial method (59.1 ±  13.7%). Despite the decrease in engorgement and hatch rates relative to direct human arm feeding, we were easily able to maintain our colony using the artificial method for ten generations, with more eggs produced than required for experimental purposes.

**Figure 3:**
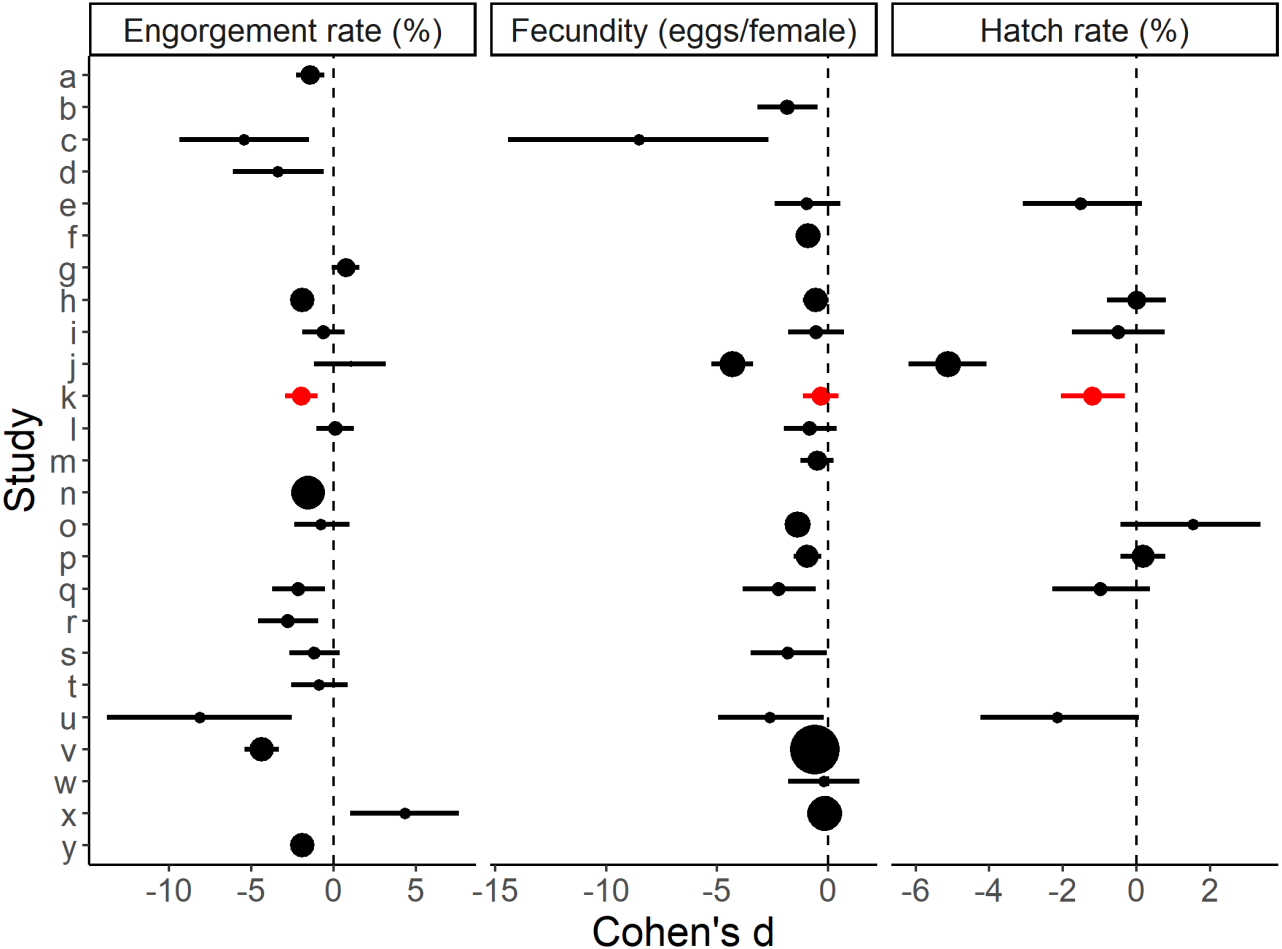
Effect sizes for blood feeding *Aedes aegypti* with an artificial method vs. a direct host method for engorgement rate, fecundity and hatch rate. Errors bars are 95% confidence intervals and the point size is the relative weight. ‘k’ refers to the present study. Study a–y (alphabetical order): a: Alto, Lounibos & Juliano, 2003, b: Bennett, 1970, c: Bunner et al., 1989, , d: Chagas et al., 2014, e: Cosgrove et al., 1994, f: Cosgrove & Wood, 1996, g: Costa-da Silva et al., 2013, h: Deng et al., 2012, i: Dhar et al., 2019, j: Dias, Bauzer & Lima, 2018, k: Faber et al., 2022 (present study), l: Finlayson, Saingamsook & Somboon, 2015, m: Harrington, Edman & Scott, 2001, n: Long et al., 2019, o: Luo, 2014, p: McMeniman, Hughes & O’Neill, 2011, q: Phasomkusolsil et al., 2013, r: Phasomkusolsil et al., 2014, s: Pina & da Fonseca, 1999, t: Pothikasikorn et al., 2007, u: Pothikasikorn et al., 2010, v: Ross, Lau & Hoffmann, 2019, w: Siria et al., 2018, x: Sri-in et al., 2020, y: Tan et al., 2016. Further methodological details of each study is given in Table S1.

The standardised effect sizes of substituting direct host feeding with an artificial method calculated from the primary literature data (Table S1) were compared with the current study. After screening, 24 studies with usable data were selected. Eighteen of these provided engorgement rates, 17 provided information on fecundity and eight provided hatch rates. The effects of the artificial blood feeding method on engorgement rate, fecundity and hatch rate observed in our study fell within the range of effects observed by other published studies (Fig. 3).

**Table 1:** Comparison of blood feeding method on engorgement and hatch rate (generalised linear model with quasibinomial distribution), and fecundity (generalised linear model with quasiPoisson distribution). Values are means ± SD, *n* = 12 for each treatment. See Table S2 for GLM regression estimates.

**Measure**	**Human**	**Skitosnack**	**Statistical test, result**
Engorgement rate (%)	97.75 ± 4.3	64.08 ± 23.2	GLM (quasibinomial), *t* = − 4.03, *P* = 5.59e−4
Fecundity (eggs/female)	56.08 ± 26.4	47.08 ± 24.7	GLM (quasiPoisson), *t* = − 0.579, *P* = 5.69e−1
Hatch rate (%)	75.17 ± 12.1	59.08 ± 13.7	GLM (quasibinomial), *t* = − 3.03, *P* = 6.13e−3

We estimate that the equipment required to blood feed a colony of mosquitoes has a cost less than $100 USD (Table 2), which includes an in-house constructed water bath and four blood feeding units capable of maintaining a colony of ∼300–400 female mosquitoes.

**Table 2:** Approximate cost of blood feeding apparatus, including water bath.

**Item**	**Approximate Cost (USD)**
*Bloodfeeding unit*	
Skirted 50 mL centrifuge tube	0.23
PharMed BPT N8F-51 silicon tubing (20 cm)	4.51
Cable ties	0.07
Parafilm ‘M’ membrane (3 cm)	0.01
*Water bath*	
Aquarium pump	10.78
Inkbird ITC-308	31.94
2000 W element	9.90
9 L bucket	3.02
Connecting silicone tube 4.8 mm ID × 9.5 mm OD (50 cm)	0.28
**Total (with 4 bloodfeeding units)**	78.39

## Discussion

Much scientific benefit has been derived from rearing hematophagous insects in the laboratory. The safety and ethical considerations of directly feeding on human volunteers (Harrington, Foy & Bangs, 2020) and the costs and ethical considerations of directly feeding on animals (Benedict & Dotson, 2007), mean, however, that replacement methods are highly desirable. Commercial artificial blood feeding products typically have high costs (*e.g.*, ∼$2500 USD for a comparable Hemotek system) and are only sold by a limited number of suppliers which render them difficult to obtain or unavailable for some laboratories. To replace direct host feeding methods, an artificial method must be effective, reliable, easily operated and broadly accessible. Our results indicate that the setup and approach described here is as efficacious as other artificial methods when compared with direct host feeding, yet the total costs are substantially lower than commercial options. Variability between the artificial blood feeding methods in the studies we reviewed indicates the lack of a standard artificial blood feeding approach (see Table S1 for methodological details of each comparison). In many cases, financial constraints and the local availability of materials and blood may dictate which membrane material, heating method and blood source is used, yet a low-cost blood feeding method for mosquitoes has not yet been broadly adopted for research. Using inexpensive and easily available materials and a simple construction may help to standardise methodological variability between studies from research laboratories which cannot rely on commercial options. It may also be effective in doing so more generally.

Engorgement rates for artificial membrane feeding vary widely in the literature. For instance, (Ross, Lau & Hoffmann, 2019) observed engorgement rates of around 20%, and (Siria et al., 2018) observed remarkably high rates of 100%. The differences in engorgement rates between studies may be a result of various factors, including blood source, membrane material and heating method which vary widely (Table S1). Direct feeding on a host usually shows higher rates of engorgement than artificial membrane feeding for *Aedes aegypti* (Bunner et al., 1989; Pina & da Fonseca, 1999; Alto, Lounibos & Juliano, 2003; Pothikasikorn et al., 2007; Pothikasikorn et al., 2010; Deng et al., 2012; Phasomkusolsil et al., 2013; Chagas et al., 2014; Luo, 2014; Phasomkusolsil et al., 2014; Tan et al., 2016; Dhar et al., 2019; Long et al., 2019; Ross, Lau & Hoffmann, 2019). Therefore, it is evident that there are important factors influencing engorgement rates which are only present when using live animals. Host-seeking and preference in mosquitoes is complex and variable, potentially involving numerous senses including vision, hearing, mechanoreception and chemoreception (Bowen, 1991) as well as thermo- and hygrosensation (Wolff & Riffell, 2018). These behaviours are highly adaptable and can be influenced by learning (Vinauger et al., 2018; Wolff & Riffell, 2018) and selection, having genetic determinants (Takken & Verhulst, 2013). As the history of mosquito strains used in experiments is not often given in the literature, some of the comparative studies investigated in our meta-analysis may have used mosquitoes adapted over multiple generations, increasing the efficiency of the method. For example, (Ross, Lau & Hoffmann, 2019) showed that adapting *Aedes aegypti* colonies to membrane feeding over 12 generations increased their engorgement rates by 138% when using membrane feeders. We did not conduct any analyses to determine differences between generations and expect that a different experimental design with a larger number of replicates and generations would be required to observe any such effects.

When landing on the human forearm, we observed that mosquitoes usually began feeding immediately and this contrasted with an extended period of probing usually observed on the membrane feeders which did not always result in successful feeding and engorgement. This behaviour may be related to olfaction/gustation or an inability of the mosquitoes to pierce the stretched Parafilm ‘M’ membrane. When this behaviour was observed by Ross, Lau & Hoffmann (2019), they suggested that membrane feeding could be improved by choosing membrane materials that are more easily pierced. Alternative membranes were not tested in the present study, however it seems likely that collagen or other organic membranes could be used if secured to the base of the reservoir by using a strip of Parafilm ‘M’ and may increase the effectiveness of blood feeding. Other research has found that membrane feeding rates in mosquitoes could be improved through application of CO_2_ or host odour in the form of sweaty socks (Bunner et al., 1989; Andreasen et al., 2004). Before feeding, we deprived mosquitoes of sucrose solution, a source of carbohydrates and moisture, and it is likely that modifying this period may also influence engorgement rates, as dehydration level has been shown to effect blood feeding (Hagan et al., 2018). Apart from adapting colonies over several generations, we suggest that alternative membrane materials, specific olfactory cues, and deprivation of moisture could improve the engorgement rates seen with this method.

We found that fecundity was not significantly different between the artificial method and direct feeding. When comparing artificial and direct host feeding methods across studies, fecundity is usually lower in the artificial method, although the measure is highly variable and the difference is not always significant (Bennett, 1970; Bunner et al., 1989; Cosgrove et al., 1994; Cosgrove & Wood, 1996; Pina & da Fonseca, 1999; Harrington, Edman & Scott, 2001; Pothikasikorn et al., 2010; McMeniman, Hughes & O’Neill, 2011; Deng et al., 2012; Phasomkusolsil et al., 2013; Luo, 2014; Finlayson, Saingamsook & Somboon, 2015; Dias, Bauzer & Lima, 2018; Siria et al., 2018; Dhar et al., 2019; Ross, Lau & Hoffmann, 2019; Sri-in et al., 2020). Reduced fecundity may be related to nutrition (Dimond et al., 1956) or the amount of blood meal imbibed. For example, (Harrington, Edman & Scott, 2001) found *Aedes aegypti* that fed directly on hosts imbibed larger blood meals than artificially fed mosquitoes. After our blood feeding trials, each adult female mosquito was classed as either engorged or not, without accounting for partial engorgement. Mosquitoes fed on the human forearm typically appeared fatter than those fed on SkitoSnack suggesting that further studies should weigh engorged females to measure the quantity of blood meal imbibed and determine how this affects fecundity.

Hatch rates are known to differ with blood source (Phasomkusolsil et al., 2013; Dias, Bauzer & Lima, 2018; Paris et al., 2018) with egg viability being highly sensitive to parent nutrition (Gonzales & Hansen, 2016). Only nine of the 24 studies we reviewed used blood from the same species of animal to compare a direct host method with an artificial method (Table S1) and in most of these cases the blood was from a different source and had been purchased from a supplier rather than extracted from the same host as the direct feeding and therefore had been treated differently and might have been of a different age. This may be important, for example, (Pothikasikorn et al., 2007; Baughman et al., 2017) found that human blood loses its effectiveness in feeding mosquitoes after only a few weeks of refrigeration. Undoubtedly, blood source or type influenced the hatch rate results of many of the studies we reviewed and our chosen diet was likely responsible for the decreased hatch rates we observed in the present study. Work towards developing artificial blood meals for mosquitoes (Kogan, 1990; Talyuli et al., 2015; Gonzales & Hansen, 2016; Gonzales et al., 2018) may help to encourage studies which elucidate the influences of nutrition on hatch rates.

For all our validation trials of the artificial blood feeder, we used SkitoSnack, an artificial blood meal designed for mosquitoes (Gonzales et al., 2018), but the method would be equally well suited to defibrinated animal or human blood. The major advantage of SkitoSnack over natural blood is that it can be stored indefinitely as a powder at −20 °C and hydrated when required, presenting a major advantage in settings where obtaining, transporting, storing and handling fresh blood is logistically difficult. Also, unlike what has been reported for real blood (Wade, 1976; Pina & da Fonseca, 1999; Finlayson, Saingamsook & Somboon, 2015), we observed no evidence of settling or sedimentation in the membrane feeders and so it did not require mixing to ensure the quality of imbibed blood meal remained consistent during the feeding period. Ideally, any artificial diet should be consistent and chemically defined, as this enables direct testing of physiology and nutritional requirements (Kogan, 1990; Piper et al., 2014; Talyuli et al., 2015). Being of a consistent formulation, SkitoSnack has the advantage of enabling standardised blood feeding when this is a requirement of an experiment. In the present study, we assessed three general measures of blood feeding method effectiveness, however there may be other phenotypic changes not considered and these are likely linked to nutrition provided by the diet. Studies have established the viability of using SkitoSnack for long term maintenance of colonies with no effects on life history traits or fitness (Gonzales et al., 2018; Kandel et al., 2020), but as far as we are aware, the present study is the first comparison between direct human feeding and membrane feeding of *Aedes aegypti* with this diet that has been published and so provides a baseline for those seeking the advantages offered by a method and blood source which are artificial.

## Conclusions

Given the specialisations of *Aedes aegypti*, including highly sensitive senses and host seeking behaviours, its fast generation time, and the fact that its larvae are active, easily handled and exhibit consistent behaviours, it offers much value as a model organism, both for disease vectors and more generally. A reference genome now exists for *Aedes aegypti* (Nene et al., 2007; Matthews et al., 2018), alongside genome editing tools *e.g.*, (DeGennaro et al., 2013; Dong et al., 2015), as well as GAL4/UAS and Q-system transgene binary expression systems (Kokoza & Raikhel, 2011; Matthews, Younger & Vosshall, 2019), enabling a broad field of research to develop. As an emerging model organism, the methods used to culture and experiment with *Aedes aegypti* should be relatively easy and accessible. By standardising the methodology and removing the risks and ethical considerations of blood feeding (Benedict & Dotson, 2007; Achee et al., 2015), the status of *Aedes aegypti* as a model organism could be elevated, potentially providing new, generalizable insights.

Compared with traditional methods of blood feeding, artificial systems provide a simple and effective way to avoid the risks and ethical considerations associated with employing volunteers or animals. As well as being much cheaper than commercially available options, the system described here has several advantages over other methods. We have found that it is simple, robust and unlikely to break or malfunction, uses a small amount of blood or blood substitute (∼3 mL) and does not require ongoing attention to maintain an optimum temperature during operation, making it suitable for blood feeding assays where constant temperature is required. Being modular and flexible to any number of units means that it can be customised for a broad range of applications where the blood feeding of mosquitoes is required, without the need for serious redesign. In experiments where live virus or other pathogens are used to infect mosquitoes, contaminated components (the modified centrifuge tube) could, after use, be autoclaved or discarded without much concern for cost. Finally, electing a standardised method of blood feeding mosquitoes by using well described, commonly available materials as well as an artificial blood meal of known and consistent quality may help to enable comparisons between experimental research trials which might otherwise be confounded.

##  Supplemental Information

10.7717/peerj.14247/supp-1 Supplemental Information 1 Membrane type, blood type, animal host and heating method of studies used in review * present study.

10.7717/peerj.14247/supp-2 Supplemental Information 2 Estimated GLM regression parameters for hatch rate and engorgement rate (quasibinomial) and fecundity (quasiPoisson)

10.7717/peerj.14247/supp-3 Supplemental Information 3 Video detailing construction and operation of the blood feeding system

## Additional Information and Declarations

### Data Availability

The following information was supplied regarding data availability: The script and raw data are available at Figshare: - The script to analyse bloodfeeding data: Faber, Peter (2020): BF analysis script. Monash University. Dataset. https://doi.org/10.26180/13141298
- The raw data for bloodfeeding experiments: Faber, Peter (2020): BF_rawdata.csv. Monash University. Dataset. https://doi.org/10.26180/13121657
- The data used for metaanalysis: Faber, Peter (2020): BF_metaanalysis. Monash University. Dataset. https://doi.org/10.26180/13141286.

## References

[ref-1] Achee NL, Youngblood L, Bangs MJ, Lavery JV, James S (2015). Considerations for the use of human participants in vector biology research: a tool for investigators and regulators. Vector-Borne and Zoonotic Diseases.

[ref-2] Alto BW, Lounibos LP, Juliano SA (2003). Age-dependent bloodfeeding of Aedes aegypti and Aedes albopictus on artificial and living hosts. Journal of the American Mosquito Control Association.

[ref-3] Alves FP, Gil LHS, Marrelli MT, Ribolla PE, Camargo EP, Da Silva LHP (2005). Asymptomatic carriers of Plasmodium spp. as infection source for malaria vector mosquitoes in the Brazilian Amazon. Journal of Medical Entomology.

[ref-4] Andreasen M, Birtles A, Curtis C, Wood R (2004). Enhanced blood feeding of Anopheles mosquitoes (Diptera: Culicidae) through membranes with applied host odour. Bulletin of Entomological Research.

[ref-5] Baglan H, Lazzari C, Guerrieri F (2017). Learning in mosquito larvae (Aedes aegypti): habituation to a visual danger signal. Journal of Insect Physiology.

[ref-6] Bailey DL, Dame DA, Munroe WL, Thomas JA (1978). Colony maintenance of Anopheles albimanus Wiedemann by feeding preserved blood through natural membrane. Mosq News.

[ref-7] Baughman T, Peterson C, Ortega C, Preston SR, Paton C, Williams J, Guy A, Omodei G, Johnson B, Williams H, O’Neill SL, Ritchie SA, Dobson SL, Madan D (2017). A highly stable blood meal alternative for rearing Aedes and Anopheles mosquitoes. PLOS Neglected Tropical Diseases.

[ref-8] Benedict M, Dotson E (2007). Methods in Anopheles research. 2015.

[ref-9] Bennett GF (1970). The influence of the blood meal type on the fecundity of Aedes (Stegomyia) aegypti L.(Diptera: Culicidae). Canadian Journal of Zoology.

[ref-10] Bowen M (1991). The sensory physiology of host-seeking behavior in mosquitoes. Annual Review of Entomology.

[ref-11] Brady OJ, Gething PW, Bhatt S, Messina JP, Brownstein JS, Hoen AG, Moyes CL, Farlow AW, Scott TW, Hay SI (2012). Refining the global spatial limits of dengue virus transmission by evidence-based consensus. PLOS Neglected Tropical Diseases.

[ref-12] Bunner BL, Scott RL, Dobson SE, Anderson LM, Boobar LR (1989). Comparison of artificial membrane with live host bloodfeeding of Aedes aegypti (L.)(Diptera: Culicidae). Journal of Entomological Science.

[ref-13] Chagas AC, Ramirez JL, Jasinskiene N, James AA, Ribeiro JM, Marinotti O, Calvo E (2014). Collagen-binding protein, Aegyptin, regulates probing time and blood feeding success in the dengue vector mosquito, Aedes aegypti. Proceedings of the National Academy of Sciences of the United States of America.

[ref-14] Clemons A, Haugen M, Flannery E, Tomchaney M, Kast K, Jacowski C, Le C, Mori A, Holland WS, Sarro J (2010). Aedes aegypti: an emerging model for vector mosquito development. Cold Spring Harbor Protocols.

[ref-15] Cosgrove JB, Wood RJ (1996). Effects of variations in a formulated protein meal on the fecundity and fertility of female mosquitoes. Medical and Veterinary Entomology.

[ref-16] Cosgrove J, Wood R, Petrić D, Evans D, Abbott R (1994). A convenient mosquito membrane feeding system. Journal of the American Mosquito Control Association.

[ref-17] Costa-da Silva AL, Carvalho DO, Kojin BB, Capurro ML (2014). Implementation of the artificial feeders in hematophagous arthropod research cooperates to the vertebrate animal use replacement, reduction and refinement (3Rs) principle. Journal of Clinical Research & Bioethics.

[ref-18] Costa-da Silva AL, Navarrete FR, Salvador FS, Karina-Costa M, Ioshino RS, Azevedo DS, Rocha DR, Romano CM, Capurro ML (2013). Glytube: a conical tube and parafilm M-based method as a simplified device to artificially blood-feed the dengue vector mosquito, Aedes aegypti. PLOS ONE.

[ref-19] Crawley MJ (2012). The R book.

[ref-20] DeGennaro M, McBride CS, Seeholzer L, Nakagawa T, Dennis EJ, Goldman C, Jasinskiene N, James AA, Vosshall LB (2013). orco mutant mosquitoes lose strong preference for humans and are not repelled by volatile DEET. Nature.

[ref-21] Degner EC, Harrington LC (2016). A mosquito sperm’s journey from male ejaculate to egg: mechanisms, molecules, and methods for exploration. Molecular Reproduction and Development.

[ref-22] Deng L, Koou S, Png A, Ng L, Lam-Phua S (2012). A novel mosquito feeding system for routine blood-feeding of Aedes aegypti and Aedes albopictus. Tropical Biomedicine.

[ref-23] Dhar I, Akther T, Bashar K, Tabassum S, Howlader AJ, Munshi SU (2019). Development of a cheap and simple artificial feeding device for studying dengue virus transmission in Aedes aegypti mosquito at the resource-poor setups. International Journal of Mosquito Research.

[ref-24] Dias LDS, Bauzer LGSDR, Lima JBP (2018). Artificial blood feeding for Culicidae colony maintenance in laboratories: does the blood source condition matter?. Revista do Instituto de Medicina Tropical de São Paulo.

[ref-25] Dimond J, Lea A, Hahnert W, DeLong D (1956). The amino acids required for egg production in Aedes aegypti. The Canadian Entomologist.

[ref-26] Dong S, Lin J, Held NL, Clem RJ, Passarelli AL, Franz AW (2015). Heritable CRISPR/Cas9-mediated genome editing in the yellow fever mosquito, Aedes aegypti. PLOS ONE.

[ref-27] Du S, Liu Y, Liu J, Zhao J, Champagne C, Tong L, Zhang R, Zhang F, Qin C-F, Ma P (2019). Aedes mosquitoes acquire and transmit Zika virus by breeding in contaminated aquatic environments. Nature Communications.

[ref-28] Duong V, Lambrechts L, Paul RE, Ly S, Lay RS, Long KC, Huy R, Tarantola A, Scott TW, Sakuntabhai A (2015). Asymptomatic humans transmit dengue virus to mosquitoes. Proceedings of the National Academy of Sciences of the United States of America.

[ref-29] Edman JD, Scott TW (1987). Host defensive behaviour and the feeding success of mosquitoes. International Journal of Tropical Insect Science.

[ref-30] Feachem RG, Phillips AA, Hwang J, Cotter C, Wielgosz B, Greenwood BM, Sabot O, Rodriguez MH, Abeyasinghe RR, Ghebreyesus TA (2010). Shrinking the malaria map: progress and prospects. The Lancet.

[ref-31] Finlayson C, Saingamsook J, Somboon P (2015). A simple and affordable membrane-feeding method for Aedes aegpyti and Anopheles minimus (Diptera: Culicidae). Acta Tropica.

[ref-32] Foster WA (1980). Colonization and maintenance of mosquitoes in the laboratory. Pathology, vector studies, and culture.

[ref-33] Gonzales KK, Hansen IA (2016). Artificial diets for mosquitoes. International Journal of Environmental Research and Public Health.

[ref-34] Gonzales KK, Rodriguez SD, Chung H-N, Kowalski M, Vulcan J, Moore EL, Li Y, Willette SM, Kandel Y, Van Voorhies WA (2018). The effect of SkitoSnack, an artificial blood meal replacement, on Aedes aegypti life history traits and gut microbiota. Scientific Reports.

[ref-35] Graumans W, Heutink R, van Gemert G-J, vandeVegte Bolmer M, Bousema T, Collins KA (2020). A mosquito feeding assay to examine Plasmodium transmission to mosquitoes using small blood volumes in 3D printed nano-feeders. Parasites & Vectors.

[ref-36] Gubler DJ (1998). Dengue and dengue hemorrhagic fever. Clinical Microbiology Reviews.

[ref-37] Gubler DJ (2002). The global emergence/resurgence of arboviral diseases as public health problems. Archives of Medical Research.

[ref-38] Gunathilaka N, Ranathunge T, Udayanga L, Abeyewickreme W (2017). Efficacy of blood sources and artificial blood feeding methods in rearing of Aedes aegypti (Diptera: Culicidae) for sterile insect technique and incompatible insect technique approaches in Sri Lanka. BioMed Research International.

[ref-39] Hagan RW, Didion EM, Rosselot AE, Holmes CJ, Siler SC, Rosendale AJ, Hendershot JM, Elliot KS, Jennings EC, Nine GA (2018). Dehydration prompts increased activity and blood feeding by mosquitoes. Scientific Reports.

[ref-40] Hamman EA, Pappalardo P, Bence JR, Peacor SD, Osenberg CW (2018). Bias in meta-analyses using Hedges’d. Ecosphere.

[ref-41] Harrington LC, Edman JD, Scott TW (2001). Why do female Aedes aegypti (Diptera: Culicidae) feed preferentially and frequently on human blood?. Journal of Medical Entomology.

[ref-42] Harrington LC, Foy BD, Bangs MJ (2020). Considerations for human blood-feeding and Arthropod exposure in vector biology research: an essential tool for investigations and disease control. Vector-Borne and Zoonotic Diseases.

[ref-43] Haugen M, Flannery E, Tomchaney M, Mori A, Behura SK, Severson DW, Duman-Scheel M (2011). Semaphorin-1a is required for Aedes aegypti embryonic nerve cord development. PLOS ONE.

[ref-44] Hedges LV (1981). Distribution theory for Glass’s estimator of effect size and related estimators. journal of Educational Statistics.

[ref-45] Hemotek (2020). Hemotek membrane feeding systems for blood sucking insects. http://hemotek.co.uk/.

[ref-46] Higgins JP, Thomas J, Chandler J, Cumpston M, Li T, Page MJ, Welch VA (2019). Cochrane handbook for systematic reviews of interventions.

[ref-47] Kandel Y, Mitra S, Jimenez X, Rodriguez SD, Romero A, Blakely BN, Cho SY, Pelzman C, Hansen IA (2020). Long-Term Mosquito culture with SkitoSnack, an artificial blood meal replacement. PLOS Neglected Tropical Diseases.

[ref-48] Kelley K (2007). Confidence intervals for standardized effect sizes: Theory, application, and implementation. Journal of Statistical Software.

[ref-49] Kelley K (2015).

[ref-50] Knols BG, Njiru BN, Mathenge EM, Mukabana WR, Beier JC, Killeen GF (2002). MalariaSphere: a greenhouse-enclosed simulation of a natural Anopheles gambiae (Diptera: Culicidae) ecosystem in western Kenya. Malaria Journal.

[ref-51] Kogan P (1990). Substitute blood meal for investigating and maintaining Aedes aegypti (Diptera: Culicidae). Journal of Medical Entomology.

[ref-52] Kokoza VA, Raikhel AS (2011). Targeted gene expression in the transgenic Aedes aegypti using the binary Gal4-UAS system. Insect Biochemistry and Molecular Biology.

[ref-53] Lequime S, Paul RE, Lambrechts L (2016). Determinants of Arbovirus Vertical transmission in mosquitoes. PLOS Pathogens.

[ref-54] Long KC, Sulca J, Bazan I, Astete H, Jaba HL, Siles C, Kocher C, Vilcarromero S, Schwarz J, Escobedo-Vargas KS (2019). Feasibility of feeding Aedes aegypti mosquitoes on dengue virus-infected human volunteers for vector competence studies in Iquitos, Peru. PLOS Neglected Tropical Diseases.

[ref-55] Luo YP (2014). A novel multiple membrane blood-feeding system for investigating and maintaining Aedes aegypti and Aedes albopictus mosquitoes. Journal of Vector Ecology.

[ref-56] Matthews BJ, Dudchenko O, Kingan SB, Koren S, Antoshechkin I, Crawford JE, Glassford WJ, Herre M, Redmond SN, Rose NH (2018). Improved reference genome of Aedes aegypti informs arbovirus vector control. Nature.

[ref-57] Matthews BJ, Vosshall LB (2020). How to turn an organism into a model organism in 10 ‘easy’steps. Journal of Experimental Biology.

[ref-58] Matthews BJ, Younger MA, Vosshall LB (2019). The ion channel ppk301 controls freshwater egg-laying in the mosquito Aedes aegypti. Elife.

[ref-59] McMeniman CJ, Hughes GL, O’Neill SL (2011). A Wolbachia symbiont in Aedes aegypti disrupts mosquito egg development to a greater extent when mosquitoes feed on nonhuman versus human blood. Journal of Medical Entomology.

[ref-60] Musso D, Rodriguez-Morales AJ, Levi JE, Cao-Lormeau V-M, Gubler DJ (2018). Unexpected outbreaks of arbovirus infections: lessons learned from the Pacific and tropical America. The Lancet Infectious Diseases.

[ref-61] Ndebele P, Musesengwa R (2012). Ethical dilemmas in malaria vector research in Africa: making the difficult choice between mosquito, science and humans. Malawi Medical Journal.

[ref-62] Nene V, Wortman JR, Lawson D, Haas B, Kodira C, Tu ZJ, Loftus B, Xi Z, Megy K, Grabherr M (2007). Genome sequence of Aedes aegypti, a major arbovirus vector. Science.

[ref-63] Nyika A (2009). Animal research ethics in Africa: an overview. Acta Tropica.

[ref-64] O’Neill SL, Ryan PA, Turley AP, Wilson G, Retzki K, Iturbe-Ormaetxe I, Dong Y, Kenny N, Paton CJ, Ritchie SA (2018). Scaled deployment of Wolbachia to protect the community from dengue and other Aedes transmitted arboviruses. Gates Open Research.

[ref-65] Paris V, Cottingham E, Ross P, Axford J, Hoffmann A (2018). Effects of alternative blood sources on Wolbachia infected Aedes aegypti females within and across generations. Insects.

[ref-66] Phasomkusolsil S, Pantuwatana K, Tawong J, Khongtak W, Monkanna N, Kertmanee Y, Damdangdee N, McCardle PW, Schuster AL (2014). Factors influencing the feeding response of laboratory-reared Aedes aegypti. Southeast Asian Journal of Tropical Medicine and Public Health.

[ref-67] Phasomkusolsil S, Tawong J, Monkanna N, Pantuwatana K, Damdangdee N, Khongtak W, Kertmanee Y, Evans BP, Schuster AL (2013). Maintenance of mosquito vectors: effects of blood source on feeding, survival, fecundity, and egg hatching rates. Journal of Vector Ecology.

[ref-68] Pick JL, Nakagawa S, Noble DW (2019). Reproducible, flexible and high-throughput data extraction from primary literature: the metaDigitise r package. Methods in Ecology and Evolution.

[ref-69] Pina IG, da Fonseca AH (1999). Comportamento de Aedes aegypti L. 1762 (Diptera: Culicidae) alimentados artificialmente com sangue de diferentes espécies de doadores. Revista de Patologia Tropical/Journal of Tropical Pathology.

[ref-70] Piper MD, Blanc E, Leitão Gonçalves R, Yang M, He X, Linford NJ, Hoddinott MP, Hopfen C, Soultoukis GA, Niemeyer C (2014). A holidic medium for Drosophila melanogaster. Nature Methods.

[ref-71] Pothikasikorn J, Bangs M, Chareonviriyaphap T, Roongruangchai K, Roongruangchai J (2007). Comparison of blood feeding response and infection of Aedes aegypti to Wuchereria bancrofti using animal membranes and direct host contact. Journal of the American Mosquito Control Association.

[ref-72] Pothikasikorn J, Boonplueang R, Suebsaeng C, Khaengraeng R, Chareonviriyaphap T (2010). Feeding response of Aedes aegypti and Anopheles dirus (Diptera: Culicidae) using out-of-date human blood in a membrane feeding apparatus. Journal of Vector Ecology.

[ref-73] R Core Team (2018). A language and environment for statistical computing. https://www.r-project.org.

[ref-74] Romano D, Stefanini C, Canale A, Benelli G (2018). Artificial blood feeders for mosquitoes and ticks—Where from, where to?. Acta tropica.

[ref-75] Ross PA, Axford JK, Richardson KM, Endersby-Harshman NM, Hoffmann AA (2017). Maintaining Aedes aegypti mosquitoes infected with Wolbachia. JoVE (Journal of Visualized Experiments).

[ref-76] Ross PA, Lau M-J, Hoffmann AA (2019). Does membrane feeding compromise the quality of Aedes aegypti mosquitoes?. PLOS ONE.

[ref-77] Rutledge L, Ward R, Gould D (1964). Studies on the feeding response of mosquitoes to nutritive solutions in a new membrane feeder. Mosquito News.

[ref-78] Schmidt-Ott U, Lynch JA (2016). Emerging developmental genetic model systems in holometabolous insects. Current Opinion in Genetics & Development.

[ref-79] Schneider CA, Rasband WS, Eliceiri KW (2012). NIH image to ImageJ: 25 years of image analysis. Nature Methods.

[ref-80] Sciences, CL Mosquito Feeders, Membrane Style. https://chemglass.com/pages/Mosquito_Feeder_Glassware.

[ref-81] Services USDOHAH (2015). National Institutes of Health: Public Health Service Policy on Humane Care and Use of Laboratory Animals.

[ref-82] Siria DJ, E. Batista PA, Opiyo MA, Melo EF, Sumaye RD, Ngowo HS, Eiras AE, Okumu FO (2018). Evaluation of a simple polytetrafluoroethylene (PTFE)-based membrane for blood-feeding of malaria and dengue fever vectors in the laboratory. Parasites & Vectors.

[ref-83] Sri-in C, Weng S-C, Shiao S-H, Tu W-C (2020). A simplified method for blood feeding, oral infection, and saliva collection of the dengue vector mosquitoes. PLOS ONE.

[ref-84] Takken W, Verhulst NO (2013). Host preferences of blood-feeding mosquitoes. Annual Review of Entomology.

[ref-85] Talyuli OAC, Bottino-Rojas V, Taracena ML, Soares ALM, Oliveira JHM, Oliveira PL (2015). The use of a chemically defined artificial diet as a tool to study Aedes aegypti physiology. Journal of Insect Physiology.

[ref-86] Tan C-H, Wong P-SJ, Li M-ZI, Yang H-T, Chong C-S, Lee LK, Yuan S, Leo Y-S, Ng L-C, Lye DC (2016). Membrane feeding of dengue patient’s blood as a substitute for direct skin feeding in studying Aedes-dengue virus interaction. Parasites & Vectors.

[ref-87] Vinauger C, Lahondère C, Wolff GH, Locke LT, Liaw JE, Parrish JZ, Akbari OS, Dickinson MH, Riffell JA (2018). Modulation of host learning in Aedes aegypti mosquitoes. Current Biology.

[ref-88] Wade J (1976). A new design of membrane feeder incorporating an electrical blood stirring device. Annals of Tropical Medicine & Parasitology.

[ref-89] WHO (2018). World malaria report.

[ref-90] Witmer K, Sherrard-Smith E, Straschil U, Tunnicliff M, Baum J, Delves M (2018). An inexpensive open source 3D-printed membrane feeder for human malaria transmission studies. Malaria Journal.

[ref-91] Wolff GH, Riffell JA (2018). Olfaction, experience and neural mechanisms underlying mosquito host preference. Journal of Experimental Biology.

