# Supplementary material for: A standardised low-cost membrane blood-feeder for Aedes aegypti made using common laboratory materials
Source: PeerJ. 2022 Oct 28;10:e14247. doi: 10.7717/peerj.14247 (PMC9620972; doi:10.7717/peerj.14247)
Supplement: Supplemental Information 1 — * present study. [file peerj-10-14247-s001.docx]

**Supp. Table S1: Membrane type, blood type, animal host and heating method of studies used in review. * present study.**

| **Study** | **Membrane type** | **Blood type** | **Animal host** | **Heating method** |
| --- | --- | --- | --- | --- |
| Alto 2003 | Silicone | Bovine | Chicken | Heat capacity |
| Bennet 1970 | Intestine | Guinea pig | Guinea pig | Heat capacity |
| Bunner 1989 | Parafilm | Horse, sheep, rabbit | Rabbit | Water |
| Chagas 2015 | Parafilm | Human | Mouse | Water |
| Cosgrove 1994 | Collagen | Pig | Mouse | Electric |
| Cosgrove 1996 | Collagen | Pig, formulations | Mouse | Electric |
| Costa da Silva 2013 | Parafilm | Human erythrocytes | Mouse | Heat capacity |
| Deng 2011 | Collagen | Mini pig | Guinea pig | Electric |
| Dhar 2019 | Parafilm | Human | Pigeon | Water, manual |
| Dias 2018 | Parafilm | Rabbit, sheep | Guinea pig | Water |
| Faber 2022* | Parafilm | SkitoSnack | Human | Water |
| Finlayson 2015 | Parafilm | Bovine | Rat | Heat capacity |
| Harrington 2001 | Pig intestine | Human | Human | Water |
| Long 2019 | - | Human | Human | Water |
| Luo 2014 | Parafilm, sausage casing | Pig | Mouse | Water |
| McMeniman 2011 | Collagen | Mouse | Mouse | Water |
| Phasomkusolsil 2013 | Sausage casing | Guinea pig, human, sheep | Hamster | Water |
| Phasomkusolsil 2014 | Sausage casing | Sheep | Mouse | Water |
| Pina 1999 | Silicone | Human | Human | Heat capacity |
| Pothikasikorn 2007 | Chicken skin | Human | Human | Water |
| Pothikasikorn 2010 | Mouse skin | Human | Hamster | Water |
| Ross 2019 | Collagen | Human | Human | Heat capacity |
| Siria 2018 | PTFE | Bovine | Human | Water, manual |
| Sri-in 2020 | Parafilm | Sheep erythrocytes | Mouse | Heat capacity |
| Tan 2016 | Rat skin | Human | Human | Electric |
