# Supplementary material for: A standardised low-cost membrane blood-feeder for Aedes aegypti made using common laboratory materials
Source: PeerJ. 2022 Oct 28;10:e14247. doi: 10.7717/peerj.14247 (PMC9620972; doi:10.7717/peerj.14247)
Supplement: Supplemental Information 2 [file peerj-10-14247-s002.docx]

**Supp. Table S2: Estimated GLM regression parameters for hatch rate and engorgement rate (quasibinomial) and fecundity (quasipoisson)**

|  | **Estimate** | **Standard Error** | **t-value** | **p-value** |
| --- | --- | --- | --- | --- |
| *Hatch rate (%)* |  |  |  |  |
| Intercept | 1.1258 | 0.1888 | 5.963 | 5.31E-06 |
| Artificial method | -0.7664 | 0.2528 | -3.032 | 0.00613 |
| *Engorgement rate (%)* |  |  |  |  |
| Intercept | 3.7013 | 0.753 | 4.915 | 6.47E-05 |
| Artificial method | -3.1872 | 0.7906 | -4.031 | 0.000559 |
| *Fecundity (eggs/female)* |  |  |  |  |
| Intercept | 4.0560 | 0.1153 | 35.181 | 7.82e-21 |
| Artificial method | -0.1112 | 0.1920 | -0.579 | 0.569 |
|  |  |  |  |  |
